# Supplementary material for: Reconciling Mining with the Conservation of Cave Biodiversity: A Quantitative Baseline to Help Establish Conservation Priorities
Source: PLoS One. 2016 Dec 20;11(12):e0168348. doi: 10.1371/journal.pone.0168348 (PMC5173368; doi:10.1371/journal.pone.0168348)
Supplement: S1 Dataset — (ZIP) [file pone.0168348.s002.zip › Taxa/Serra Norte/SN_2013/N5S/Lista N5S-43.pdf]

CAVIDADE N5S-0043

Classe	Ordem	Fam/Outros	Gên/Outros	Espécie	S-U	U-S	Zon.
Arachnida	Acari	Asternoseiidae		sp.84	X		E
Arachnida	Acari	Laelapidae		sp.71	X		E
Arachnida	Acari	Rhagidiidae		sp.5		X	E
Arachnida	Acari	Trombiculidae		sp.39	X		E
Arachnida	Araneae	Caponiidae		jovem		X	E
Arachnida	Araneae	Pholcidae		jovem	X	X	E
Arachnida	Araneae	Salticidae		jovem	X	X	E
Arachnida	Araneae	Scytodidae	*Scytodes*	jovem	1	1	E
Arachnida	Opiliones	Cosmetidae	*Roquettea*	*singularis*	1	1	E
Arachnida	Opiliones	Stygnidae	*Protimesius*	*gracilis*	2		E
Arachnida	Pseudoscorpiones	Chthoniidae	*Pseudochthonius*	sp.1	X		E
Chilopoda	Scutigeromorpha	Pselliodidae	*Sphendononema*	*guildingii*		5	E
Entognatha	Collembola	Entomobryidae		sp.6	X		E
Entognatha	Collembola	Paronellidae		sp.1		X	E
Insecta	Blattodea	Blattidae		sp.4		1	E
Insecta	Blattodea	Polyphagidae		sp.1	1		E
Insecta	Coleoptera	Staphylinidae	Pselaphinae	sp.15	X		E
Insecta	Diptera	Cecidomyiidae	Cecidomyiinae	sp.		X	E
Insecta	Heteroptera	Dipsocoroidea		sp.		X	E
Insecta	Heteroptera	Lygaeidae		jovem		X	E
Insecta	Homoptera	Cixiidae		jovem	X		E
Insecta	Hymenoptera	Formicidae	*Brachymyrmex*	sp.1	X		E
Insecta	Hymenoptera	Formicidae	*Dolichoderus*	aff. *bispinosus*	X	X	E
Insecta	Hymenoptera	Formicidae	*Gnamptogenys*	sp.1	X	X	E
Insecta	Lepidoptera	Tineoidea		sp.3		X	E
Insecta	Orthoptera	Phalangopsidae	*Paraclodes*	sp.		3	E
Insecta	Psocoptera	Lepidopsocidae	*Lepidopsocus*	sp.1	X		E
Insecta	Psocoptera	Ptiloneuridae	*Triplocania*	sp.9	X		E
Insecta	Psocoptera			jovem	X	X	E
Insecta	Thysanura	Nicoletiidae		jovem		X	E
Malacostraca	Isopoda	Dubioniscidae		sp.1		X	E
